# Supplementary material for: Intraspecific venom variation in the medically important puff adder (Bitis arietans): Comparative venom gland transcriptomics, in vitro venom activity and immunological recognition by antivenom
Source: PLoS Negl Trop Dis. 2024 Oct 18;18(10):e0012570. doi: 10.1371/journal.pntd.0012570 (PMC11524477; doi:10.1371/journal.pntd.0012570)
Supplement: S1 Fig — Fractions from the HPLC traces shown in Fig 2 in the main text were subject to SDS-PAGE under reducing conditions and the those containing a pair of bands in the 13–18 kDa region typical of alpha and beta CLP subunits are shown [left hand gels]. These same fractions were also subjected to SDS-PAGE under non-reducing conditions [right hand gels] and most contained a strong band at 28–29 kDa corresponding to the intact heterodimeric CLP. In some minor forms [TZA 3, NGA 2 and SA 4] a 70–75 kDa protein was observed under non-reducing conditions and these are likely to be high molecular weight forms of CLPs. The SVMP peaks were identified by co-elution of the respective SVMPIIs purified in a separate study [52]. The gels used were BioRad 4–20% acrylamide, stained with Coomassie Blue R250. (DOCX) [file pntd.0012570.s001.docx]

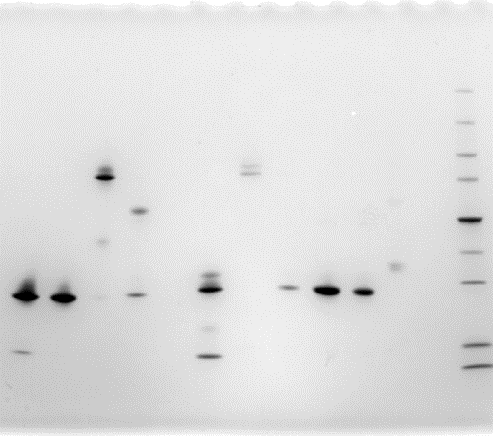

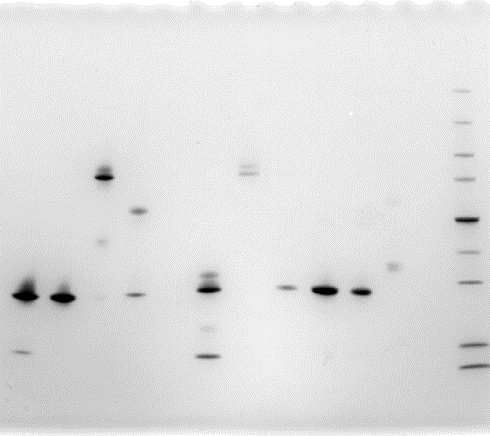


Reduced

Non-reduced

CLP 4

CLP 3

CLP 2

CLP 1

SVMPIIs


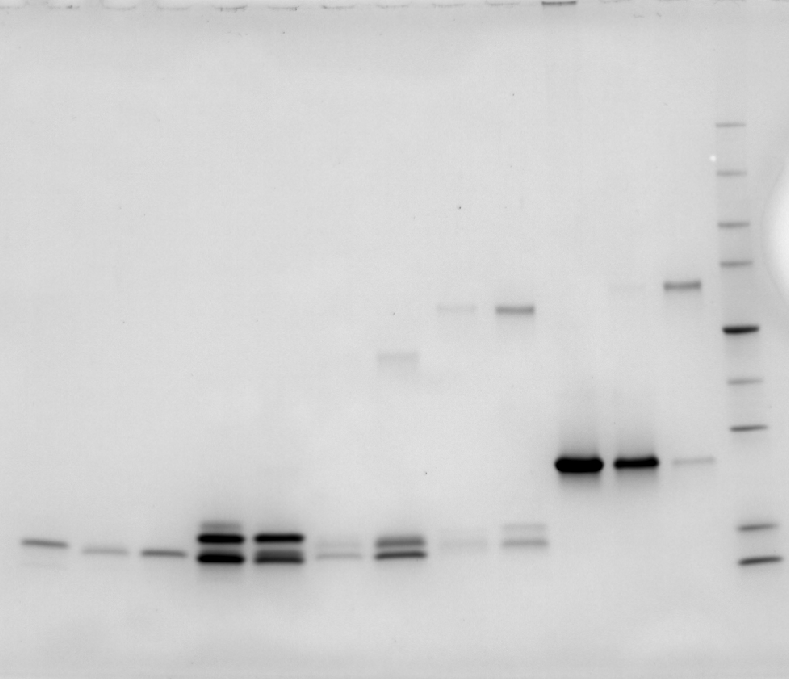

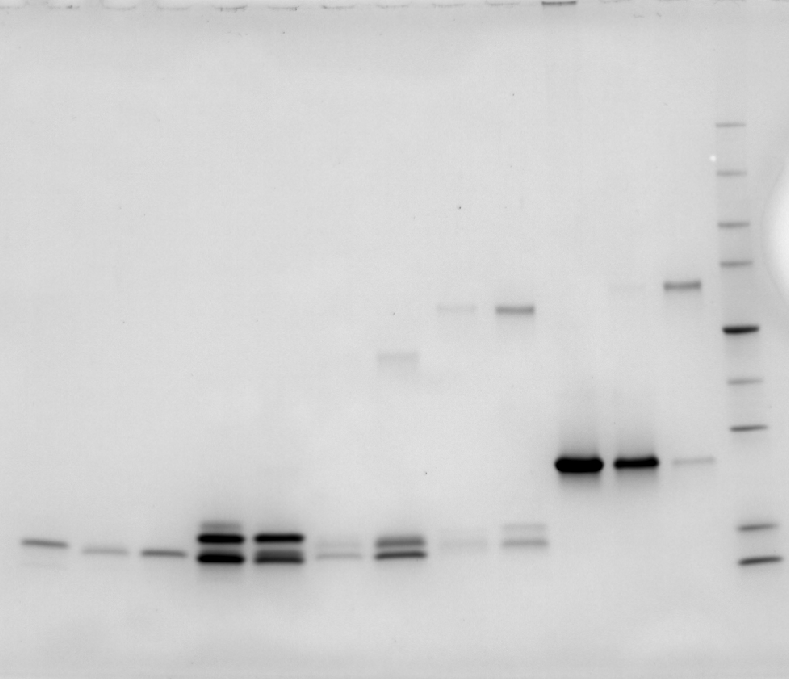

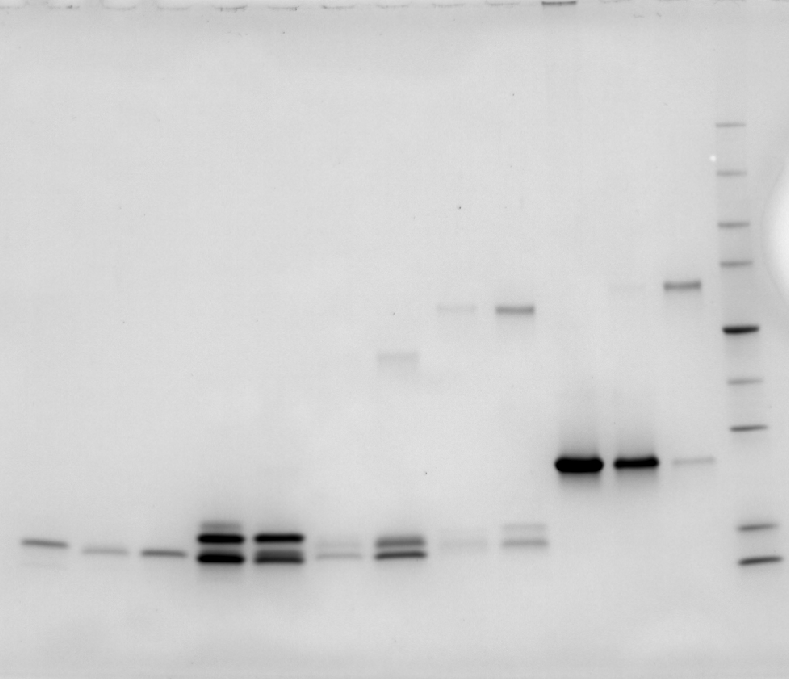

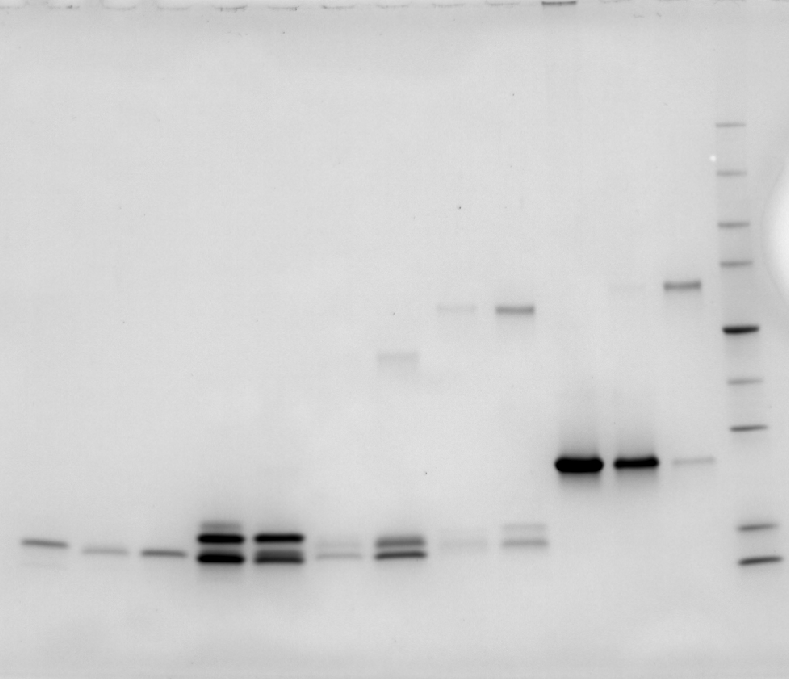


70

50

30

40

15

10

1 2 3 4 M

1 2 3 4 M


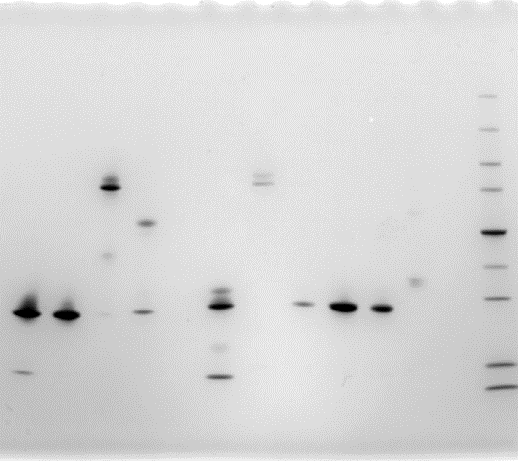

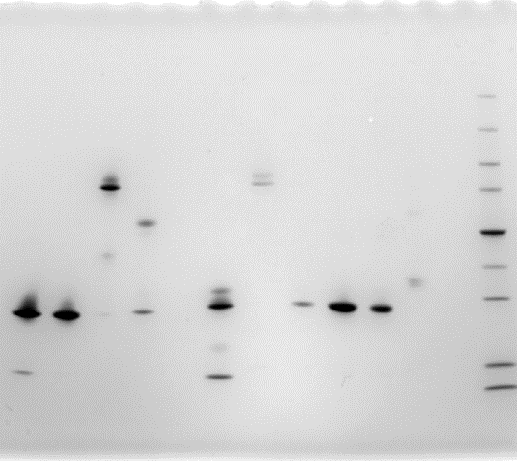


Reduced

Non-reduced

CLP 4

SVMPIIs

CLP 3


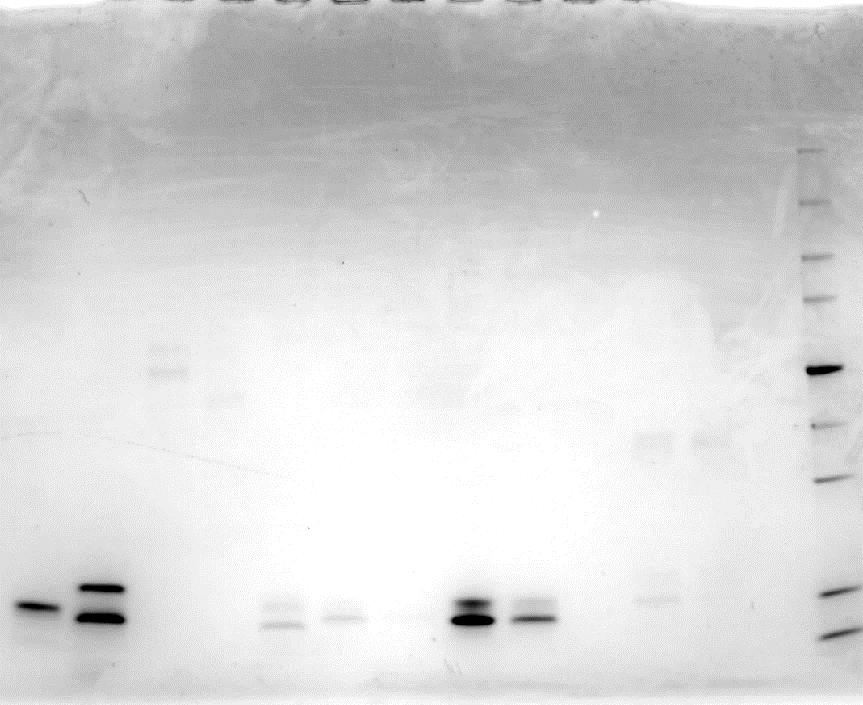

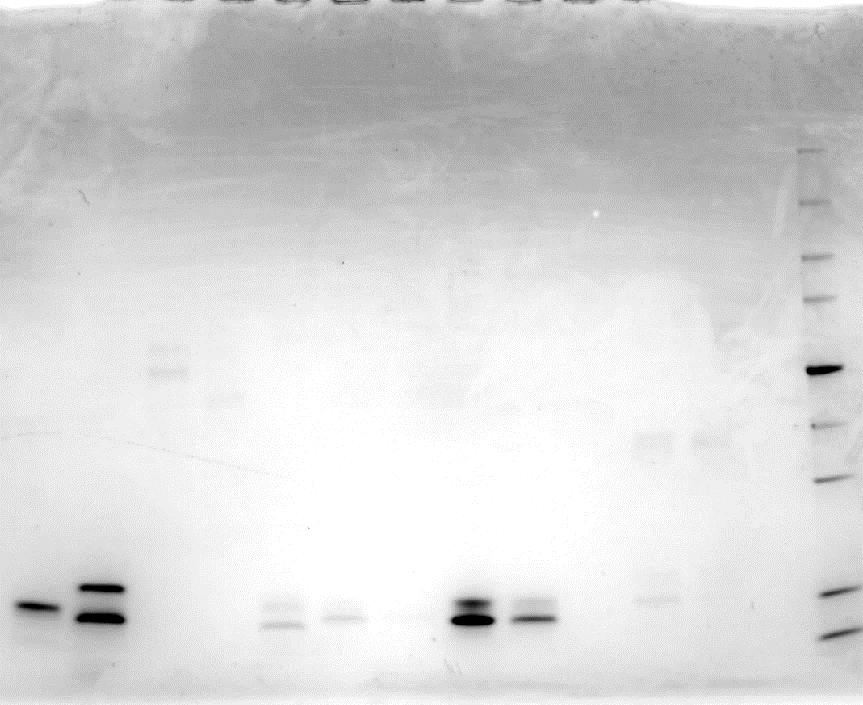

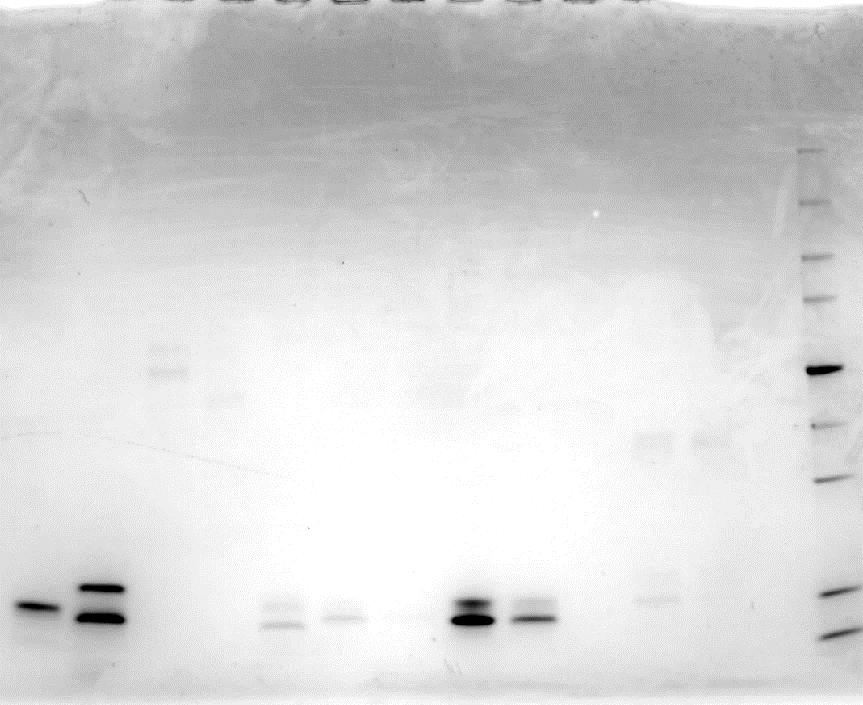

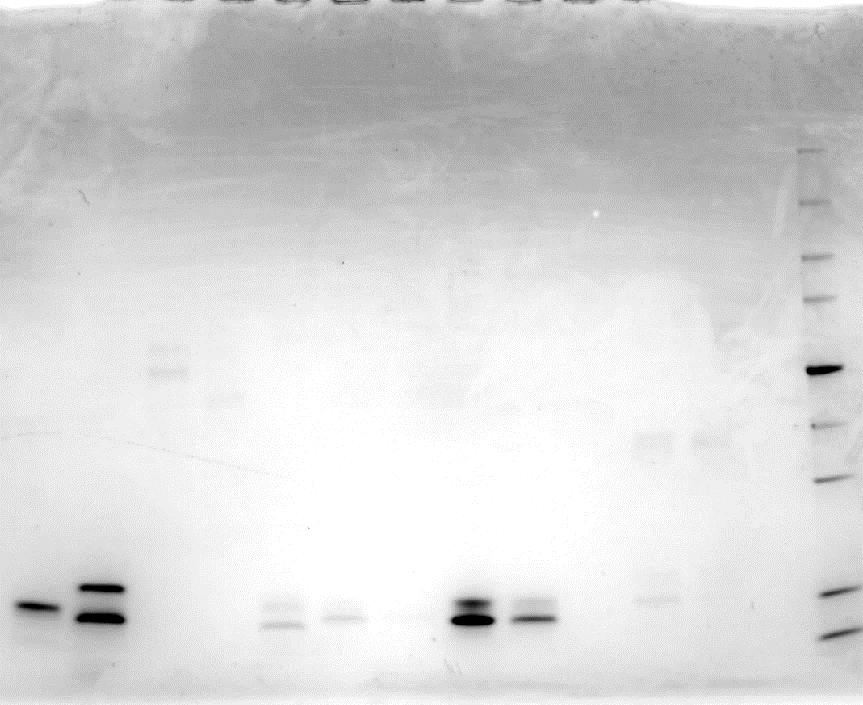


70

50

40

30

10

15

1 2 3 4 M

1 2 3 4 M


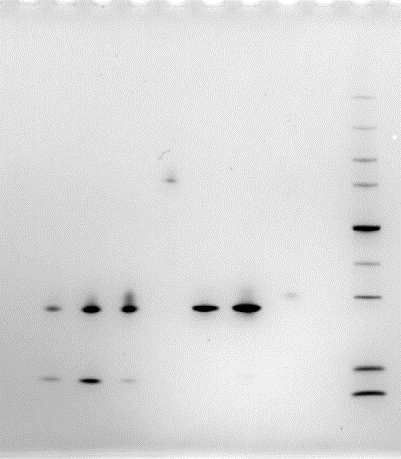


SVMPIIs

CLP 6

CLP 5

CLP 4

CLP 7

CLP 1-3

Non-reduced

Reduced


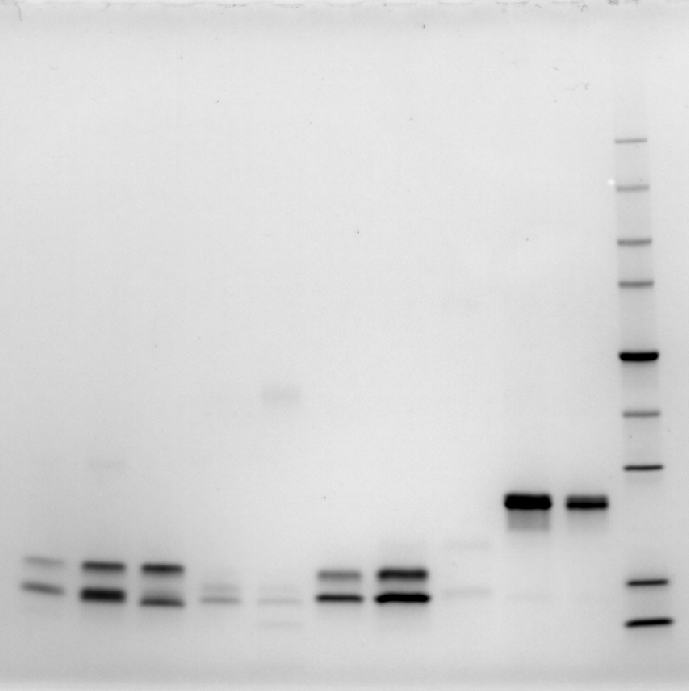

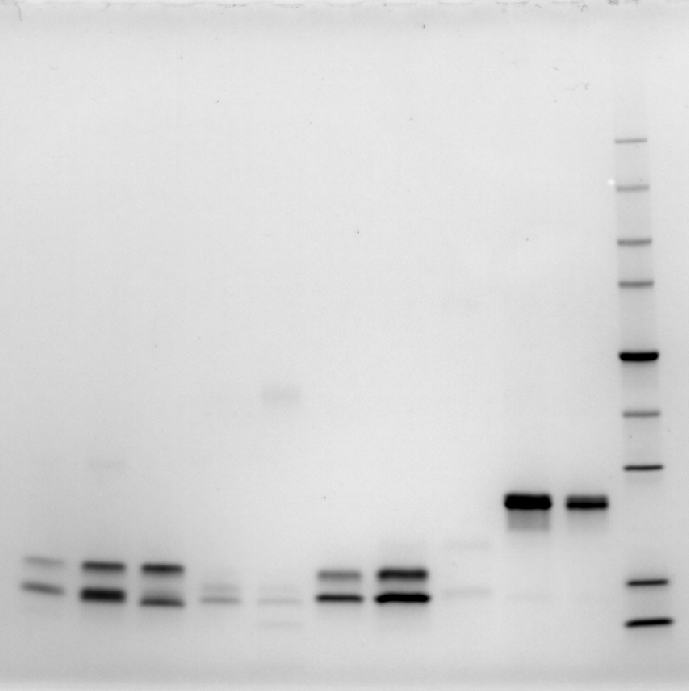

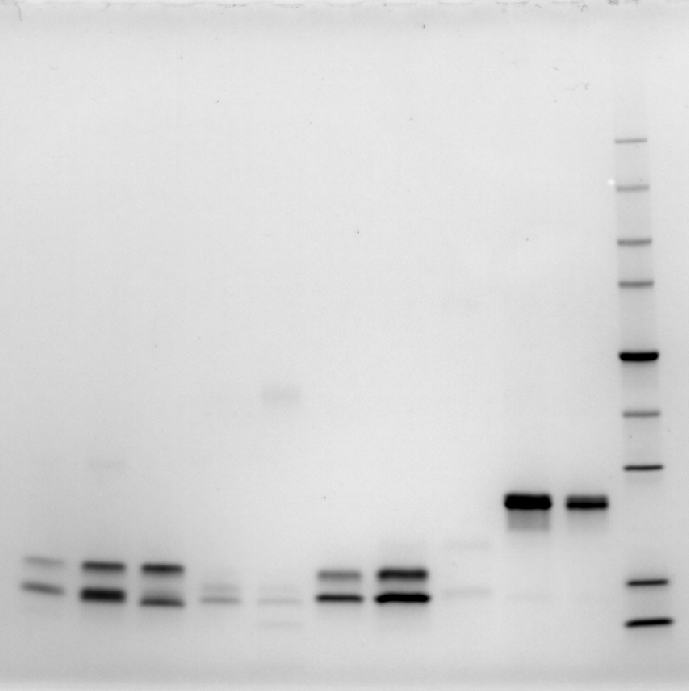


70

50

40

30

15

10

1 2 3 4 5 6 7 M

1 2 3 4 5 6 7 M

**Figure S1. SDS-PAGE analysis of the CLP-containing peaks in the RP-HPLC profiles of B. arietans venoms.** Fractions from the HPLC traces shown in Fig. 2 in the main text were subject to SDS-PAGE under reducing conditions and the those containing a pair of bands in the 13 -18 kDa region typical of alpha and beta CLP subunits are shown [left hand gels]. These same fractions were also subjected to SDS-PAGE under non-reducing conditions [right hand gels] and most contained a strong band at 28-29 kDa corresponding to the intact heterodimeric CLP. In some minor forms [TZA 3, NGA 2 and SA 4] a 70-75 kDa protein was observed under non-reducing conditions and these are likely to be high molecular weight forms of CLPs. The SVMP peaks were identified by co-elution of the respective SVMPIIs purified in a separate study (53). The gels used were BioRad 4-20% acrylamide, stained with Coomassie Blue R250.
