# Supplementary material for: Intraspecific venom variation in the medically important puff adder (Bitis arietans): Comparative venom gland transcriptomics, in vitro venom activity and immunological recognition by antivenom
Source: PLoS Negl Trop Dis. 2024 Oct 18;18(10):e0012570. doi: 10.1371/journal.pntd.0012570 (PMC11524477; doi:10.1371/journal.pntd.0012570)
Supplement: Attachment — Submitted filename: Comments.pdf [file pntd.0012570.s002.pdf]

The manuscript describes the intraspecific variability of *Bitis arietans* venom from three different locations (Nigeria, Tanzania, and South Africa), as well as the immune recognition and neutralisation of its three main enzymatic activities (SVMP, SVSP, and PLA2). The manuscript is well-grounded, justifying the relevance of studying the compositional and functional variability of venom from species with broad geographic distribution and significant medical importance. The high-quality results are well presented and discussed. I recommend publishing this manuscript in PLOS Neglected Tropical Diseases after a few minor revision, as outlined below.

1. Page 6 – lines 126-127: The authors should take into consideration the (limited) sample size used (6 specimens from Nigeria, 3 from Tanzania, and 1 from South Africa) when interpreting the results. Although this limits the interpretation at the populational level, it does not diminish the contribution or relevance of the manuscript, but should be acknowledged in the text.

2. Page 6 – lines 130-131: The antivenoms used expired in 2016/2017. Despite documented stability of expired antivenoms (as published by the authors themselves), this should be mentioned in the manuscript.

Solano G, Cunningham S, Edge RJ, Duran G, Sanchez A, Villalta M, Clare RH, Wilkinson MC, Marriott AE, Abada C, Menzies SK, Keen M, Lalloo DG, Stienstra Y, Abouyannis M, Casewell NR, León G, Ainsworth S. African polyvalent antivenom can maintain pharmacological stability and ability to neutralise murine venom lethality for decades post-expiry: evidence for increasing antivenom shelf life to aid in alleviating chronic shortages. BMJ Glob Health. 2024 Mar 13;9(3):e014813. doi: 10.1136/bmjgh-2023-014813. PMID: 38485142; PMCID: PMC10941113.

3. Page 15 – lines 334-336: "However, Nigerian *B. arietans* had a greater percentage of the total toxinome attributed to PLA2 expression compared to both Tanzanian and South African datasets (5.0% compared to 4.4% and 2.8%, respectively)". But conversely, Figure 1b shows that the percentage of 4.4% corresponds to South African *B. arietans*, while 2.8% corresponds to Tanzanian *B. arietans*. Please, correct the text or the graphic in figure 1b.

4. Page 17 – line 360: The last sentence needs to be finished. "(South Africa; 16.9% transcriptome expression, 22.5% toxin)" abundance, I suppose.

5. Page 17 – legend of figure 2: "The CLPs were identified by their characteristic electrophoretic patterns: paired bands of alpha and beta subunits at 14-18 kDa under reducing conditions, but a single band at 30-32 kDa under non-reducing conditions". HPLC fractions were submitted to SDS-PAGE? Where this analysis can be found or the results are not shown? How did the authors assign the peaks to toxin families? Since most proteomic characterization involves the analysis of the venom by HPLC using a C18 column, the use of previous HPLC profiles as reference (obtained with different columns) should be taken with caution. This should be mentioned in the manuscript.

6. Page 17 – legend of figure 2: "The position of the SVMPs was determined by comparison with that of *B. arietans* SVMPs purified and identified in a separate study (53)". SVMP peaks are indicated in the figure as "SVMPIIs". Is this correct? Or these

chromatographic peaks correspond to SVMP P-I (?), P-II and P-III, as reported by Juárez et al. (2006) and Dingwoke et al. (2021)?

Juárez P, Wagstaff SC, Oliver J, Sanz L, Harrison RA, Calvete JJ. Molecular cloning of disintegrin-like transcript BA-5A from a Bitis arietans venom gland cDNA library: a putative intermediate in the evolution of the long-chain disintegrin bitistatin. J Mol Evol. 2006 Jul;63(1):142-52. doi: 10.1007/s00239-005-0268-z. Epub 2006 Jun 16. PMID: 16786436.
Dingwoke EJ, Adamude FA, Mohamed G, Klein A, Salihu A, Abubakar MS, Sallau AB. Venom proteomic analysis of medically important Nigerian viper Echis ocellatus and Bitis arietans snake species. Biochem Biophys Rep. 2021 Nov 2;28:101164. doi: 10.1016/j.bbrep.2021.101164. PMID: 34765747; PMCID: PMC8571701.

7. Page 18 – line 377: "To determine whether intraspecific variation observed in enzymatic toxin activities,". This sentence needs to be reformulated.

8. Page 18 – legend of figure 3a: "Venoms were used at 1 μg/well". Please, correct the amount of venom used, since in page 9 – line 197 it is described that "Venoms were diluted to 0.5 mg/mL in PBS, and 1 μL added per well", resulting in 0.5 μg/well.

9. Page 19 – line 398: "To determine whether the captured protein". What do the authors mean with "captured protein"? Please, clarify.

10. Page 20 – legend of figure 4: Please, add the statistical representation at the end of the text. What mean ** and ***?

11. Page 23 – lines 462-463: Why "perhaps surprisingly"? It was clarified in the discussion section, but stated at this point raises this question.

12. Page 25 – lines 511-513: "Nigerian *B. arietans* was found to lack any expression of CRISP mRNA, which could indicate gene loss within this individual however, genome sequencing would be required to test this hypothesis". This sentence needs revision.

13. Page 26 – lines 524-526: "Whilst it was only possible to quantify the presence of CLPs and PII-SVMPs through RP-HPLC, the high prevalence of CLPs at the transcriptomic level was reflected in their abundance at the protein level". Same questions described in comments 5 and 6.

Minor points

Page 3 – line 52: Tanzanian → Tanzania

Page 20 – line 432: South Africa → South African

Page 23 – line 471: venom → venoms
