# Supplementary material for: Intraspecific venom variation in the medically important puff adder (Bitis arietans): Comparative venom gland transcriptomics, in vitro venom activity and immunological recognition by antivenom
Source: PLoS Negl Trop Dis. 2024 Oct 18;18(10):e0012570. doi: 10.1371/journal.pntd.0012570 (PMC11524477; doi:10.1371/journal.pntd.0012570)
Supplement: Attachment — Submitted filename: ResponsetoReviewers.docx [file pntd.0012570.s003.docx]

Dr. Charlotte A. Dawson

Centre for Snakebite Research &
 Interventions

Liverpool School of Tropical Medicine

Pembroke Place

Liverpool, L3 5QA, UK

[charlotte.dawson@lstmed.ac.uk](mailto:charlotte.dawson@lstmed.ac.uk)

16^th^ September 2024

Dear Professor Moura-da-Silva and Professor Gutiérrez,

Thank you for the consideration of our manuscript entitled “**Intraspecific venom variation in the medically important puff adders (*Bitis arietans*): comparative venom gland transcriptomics, *in vitro* venom activity and immunological recognition by antivenom**” and for providing feedback from reviewers. Based on the feedback from the reviewers, we have revised the manuscript in the following areas.

Reviewer 3 raised valuable comments regarding the transcriptomic analyses conducted for this study, specifically whether analyses were conducted to identify any low-quality chimeric contigs. Whilst this was not conducted in the original work, we have since used ChimeraKiller to analyse all contigs. This revealed a small number of contigs (12) that were classified as “low coverage”. These flagged sequences were then compared against proteomics data generated by Wilkinson *et al* 2024 to determine if there was evidence of these contigs within this data. Whilst proteomic evidence was found for one of the contigs, the remaining 11 sequences could not be identified within this dataset. Contigs that could not be verified within the proteomic data were removed from our analyses, and the manuscript updated to reflect this. We have updated the described methodology and results to include these chimeric analyses.

Reviewers raised number of queries regarding the HPLC based venom analyses of the three different *Bitis arietans* locales, as detailed in the legend on figure 2 and on lines 524-526. Firstly, reviewers requested information regarding the identification of CLPs on the provided HPLC traces. SDS-PAGE analyses were conducted on HPLC fractions, and CLPs identified via their characteristic pattern. These gels have now been included within the supplementary data accompanying this manuscript*.* Secondly, reviewers also requested clarification on the labelling of SVMPII peaks, and whether other SVMP classes may be within the peaks. These confirmation of the SVMPII within the annotated fractions is confirmed by the extensive analyses performed by Wilkinson *et al.,* 2024, which were conducted at the Centre for Snakebite Research and Interventions, using the same venoms that were included within this study.

Reviewers requested clarifications of some parts of the manuscript, specifically on lines 377, 389, 462-463 and 511-513, these passages have now been revised to improve clarity. A comment was raised on the inconsistency between the legend of figure 3a and then methodology described regarding the SVMP assay. These have now been amended to correctly state that venoms were used at 1 mg/mL. The legend of figure 4 has also been amended to contain the statistical denotations.

Reviewers also raised minor grammatical points (pages 3, 20 and 23) which have now been corrected.

Thank you again for your consideration of this manuscript, and to the reviewers for their careful consideration and comments. We hope that the revised manuscript sufficiently addresses the comments raised by reviewers.

Yours, on behalf of the co-authors,

Dr. Charlotte Dawson
Corresponding author,
Post doctoral research associate, Centre for Snakebite Research & Interventions
Liverpool School of Tropical Medicine, UK
